# Intrinsic Coherence Length Anisotropy in Nickelates and Some Iron-Based Superconductors

**DOI:** 10.3390/ma16124367

**Published:** 2023-06-13

**Authors:** Evgeny F. Talantsev

**Affiliations:** 1 M. N. Miheev Institute of Metal Physics, Ural Branch, Russian Academy of Sciences, 18, S. Kovalevskoy St., 620108 Ekaterinburg, Russia; evgeny.talantsev@imp.uran.ru; Tel.: +7-912-676-0374; 2 NANOTECH Centre, Ural Federal University, 19 Mira St., 620002 Ekaterinburg, Russia

**Keywords:** nickelate superconductors, iron-based superconductors, superconducting coherence length, anisotropy of characteristic length in supercondcutors

## Abstract

Nickelate superconductors, R_1−x_A_x_NiO_2_ (where R is a rare earth metal and A = Sr, Ca), experimentally discovered in 2019, exhibit many unexplained mysteries, such as the existence of a superconducting state with *T*_c_ (up to 18 K) in thin films and yet absent in bulk materials. Another unexplained mystery of nickelates is their temperature-dependent upper critical field, $B_{c2}\left(T\right)$, which can be nicely fitted to two-dimensional (2D) models; however, the deduced film thickness, $d_{sc,GL}$, exceeds the physical film thickness,$\;d_{sc}$, by a manifold. To address the latter, it should be noted that 2D models assume that $d_{sc}$ is less than the in-plane and out-of-plane ground-state coherence lengths, $d_{sc}<\xi_{ab}\left(0\right)$ and $d_{sc}<\xi_{c}\left(0\right)$, respectively, and, in addition, that the inequality $\xi_{c}\left(0\right)<\xi_{ab}\left(0\right)$ satisfies. Analysis of the reported experimental $B_{c2}\left(T\right)$ data showed that at least one of these conditions does not satisfy for R_1-x_A_x_NiO_2_ films. This implies that nickelate films are not 2D superconductors, despite the superconducting state being observed only in thin films. Based on this, here we propose an analytical three-dimensional (3D) model for a global data fit of in-plane and out-of-plane $B_{c2}\left(T\right)$ in nickelates. The model is based on a heuristic expression for temperature-dependent coherence length anisotropy: $\gamma_{\xi }\left(T\right)=\frac{\gamma_{\xi }\left(0\right)}{1-\frac{1}{a}\times \frac{T}{T_{c}}}$, where a>1 is a unitless free-fitting parameter. The proposed expression for $\gamma_{\xi }\left(T\right)$, perhaps, has a much broader application because it has been successfully applied to bulk pnictide and chalcogenide superconductors.

## 1. Introduction

High-temperature superconductivity in Nd_1-x_Sr_x_NiO_2_ was experimentally discovered by Li et al. [1] in 2019, while the first theoretical report in which the oxidation state of Ni^+^ was established to be a condition under which nickelates become similar to cuprates (created by Li et al. [1]) was published by Anisimov et al. [2] in 1999. This experimental discovery initiated further theoretical and experimental studies on R_1-x_A_x_NiO_2_ (where R is a rare earth and A = Sr, Ca) thin films [3,4,5,6,7,8,9,10,11,12,13,14,15,16,17,18,19,20,21,22,23,24,25,26,27,28,29,30,31,32,33,34,35,36,37,38,39,40,41,42,43,44,45,46,47,48,49,50] and bulk [51]. It is a widely accepted point of view that the superconducting state in nickelates is exhibited only in thin films [2,3,4,5,6,7,8,9,10,11,12,13,14,15,16,17,18,19,20,21,22,23,24,25,26,27,28,29,30,31,32,33,34,35,36,37,38,39,40,41,42,43,44,45,46,47,48,49,50,51], with a thickness of $d_{sc}≲15\;nm$. This is one of the primary mysteries in nickelate superconductors. 

Another unexplained mystery of nickelates is the temperature dependence of the upper critical field, $B_{c2}\left(T\right)$. For instance, when this fundamental field is measured for an applied field oriented in a perpendicular direction to the (00L) planes of the film (which will be designated as $B_{c2,perp}\left(T\right)$, herein), the dependence can be nicely fitted to the Ginzburg–Landau (GL) model [52]:

(1) $$B_{c2,perp}\left(T\right)=\frac{\phi_{0}}{2\pi }\frac{1}{\xi_{ab}^{2}\left(T\right)}=\frac{\phi_{0}}{2\pi }\frac{\left(1-\frac{T}{T_{c}}\right)}{\xi_{ab}^{2}\left(0\right)},$$

where $\phi_{0}$ is a superconducting flux quantum, and $\xi_{ab}\left(0\right)$ is ground state in-plane coherence length. When an external field is oriented in parallel to the (00L) planes of the film (which will be designated as $B_{c2,para}\left(T\right)$), the dependence reported by many research groups can be nicely fitted to the two-dimensional Ginzburg–Landau (2D-GL) model [52,53]:

(2) $$B_{c2,para}\left(T\right)=\frac{\phi_{0}}{2\pi }\frac{\sqrt{12}}{d_{sc,GL}}\frac{1}{\xi_{ab}\left(T\right)}=\frac{\phi_{0}}{2\pi }\frac{\sqrt{12}}{d_{sc,GL}}\frac{\sqrt{1-\frac{T}{T_{c}}}}{\xi_{ab}\left(0\right)},$$

where $d_{sc,GL}$ is the film thickness associated with the use of Equation (2) for data fit. 

This result should be interpreted as direct evidence of 2D superconductivity in nickelates, supporting the experimental observation that the superconducting state is observed only in thin films of nickelates. However, the deduced film thickness $d_{sc,GL}$ (from the $B_{c2,perp}\left(T\right)$ and $B_{c2,para}\left(T\right)$ data fit to Equations (1) and (2) [23]) by to 2–3 times exceeds the physical film thickness $d_{sc}$ [23]. Here we found that an identical problem, i.e., $d_{sc}≇d_{sc,GL}$, does exist for nearly all nickelate films for which experimental data were reported. 

To resolve this issue, here we point out that the derivation of Equation (2) [52,53] is based on the assumption that physical film thickness $d_{sc}$ is much less than the ground-state coherence length ξ(0) of the superconductor, which means that a 2D superconductor is defined by two conditions:

(3) $$d_{sc}\ll \xi_{ab}\left(0\right),$$

(4) $$d_{sc}\ll \xi_{c}\left(0\right),$$

where $\xi_{c}\left(0\right)$ is the out-of-plane coherence length. In addition, there is another hidden assumption for the derivation of Equation (2), which is:

(5) $$\xi_{c}\left(0\right)<\xi_{ab}\left(0\right).$$

As shown below, at least one of these conditions (Equations (3)–(5)) is not satisfied for the studied nickelate films. From this, we conclude that there is an incident, that $B_{c2,para}\left(T\right)$ data of nickelate films are nicely approximated by the square root of an independent variable of a two-fluid model:

(6) $$B_{c2,para}\left(T\right)\propto \sqrt{1-\frac{T}{T_{c}}},$$

and that the deeper physics behind this dependence should be determined. 

In this paper, we propose to resolve this problem by accepting that superconductivity in nickelates is a three-dimensional (3D) phenomenon and, thus, the upper critical field should be described by standard 3D Ginzburg–Landau equations:

{(7)Bc2,perp(T)=ϕ02π1ξab2(T)=ϕ02π(1−TTc)ξab2(0)                  (8)Bc2,para(T)=ϕ02π1ξc(T)1ξab(T)=ϕ02π1ξab(T)γξ(T)1ξab(T)=ϕ02πγξ(T)(1−TTc)ξab2(0)      

where $\gamma_{\xi }\left(T\right)=\frac{\xi_{ab}\left(T\right)}{\xi_{c}\left(T\right)}$ denotes the temperature-dependent coherence length anisotropy. By experimenting with many analytical functions, we found a remarkably simple and robust heuristic expression for $\gamma_{\xi }\left(T\right)$, which surprisingly enough can also be applied to iron-based superconductors:

(9) $$\gamma_{\xi }\left(T\right)=\frac{\xi_{ab}\left(T\right)}{\xi_{c}\left(T\right)}=\frac{\xi_{ab}\left(0\right)}{\xi_{c}\left(0\right)}\frac{1}{1-\frac{1}{a}\times \frac{T}{T_{c}}}=\gamma_{\xi }\left(0\right)\frac{1}{1-\frac{1}{a}\times \frac{T}{T_{c}}}=\gamma_{\xi }\left(0\right)\frac{1}{1-\frac{T}{T_{\gamma }}},$$

where a is a free-fitting parameter (varies within a narrow range of 1.2<a<2.3 for all studied superconductors), and $T_{\gamma }\equiv a\times T_{c}$. 

## 2. The Upper Critical Field Definition 

Before Equations (7)–(9) will be applied for $B_{c2}\left(T\right)$ data fit, we should clarify the definition of the $B_{c2}\left(T\right)$, because different research groups define this fundamental field using different criteria. 

In many reports on nickelates, the upper critical field, $B_{c2}\left(T\right)$, and, as a direct consequence of it, the coherence length, ξ(T), were defined/deduced from the magnetoresistance curves, R(T,B), by utilizing 50% of the normal state resistance criterion, i.e., $\frac{R\left(T\right)}{R_{norm}\left(T\right)}=0.5$ (it should be noted that some research groups [49] utilized the criterion of $\frac{R\left(T\right)}{R_{norm}\left(T\right)}\to 1.0$, which returns the most overestimated $T_{c}$ and $B_{c2}\left(T\right)$ and the most underestimated $\xi_{ab}\left(0\right)$ and $\xi_{c}\left(0\right)$ values). 

However, in direct experiments performed by Harvey et al. [35], it was shown that the diamagnetic response in nickelate films always appears at temperatures well below the zero-resistance temperature, $T_{c,zero}$ (see, for instance, Figure 2 in [35]). Because diamagnetism is an essential and unavoidable property of the superconducting state, the definition of the fundamental superconducting field (i.e., the upper critical field, $B_{c2}\left(T\right)$) at the condition at which the superconducting state does not exist (and thus neither the Abrikosov’s vortices, nor the phase coherence of the order parameter and the amplitude coherence of the order parameter exist) is incorrect. The definition by $\frac{R\left(T\right)}{R_{norm}\left(T\right)}=0.5$, or by any other similar ratios, except $\frac{R\left(T\right)}{R_{norm}\left(T\right)}\to 0$, causes many confusions, and the most notable one is the claim that the Pauli limiting field is violated in practically all thin film superconductors [36,54,55]. However, the primary reason for claimed violation is the definition of the upper critical field, $B_{c2}\left(T\right)$, by the criterion at which the superconducting state does not yet exist. 

It should be reaffirmed that because the upper critical field, $B_{c2}\left(T\right)$, is defined as the magnetic flux density at which the superconducting state collapses and the diamagnetism is an essential property of the superconducting state, the definition of the $B_{c2}\left(T\right)$ should be made based on the disappearance of the diamagnetic response or, if it is impossible to measure, by the $\frac{R\left(T\right)}{R_{norm}\left(T\right)}\to 0$ criterion. However, it should be mentioned that these definitions have been implemented in very few studies [56,57,58,59,60,61,62,63,64,65,66,67,68,69,70,71,72,73,74]. 

Based on remarkably overestimated $B_{c2}\left(T\right)$ values, defined by $\frac{R\left(T\right)}{R_{norm}\left(T\right)}=0.5$ or $\frac{R\left(T\right)}{R_{norm}\left(T\right)}\to 1$ [49,54,55,75,76,77,78], and a very broad resistive transition width in some thin film superconductors, new effects/phenomena can be claimed (for instance, the Pauli limiting field violation [36,54,55,75,76,77,78]). However, these new effects/phenomena can be explained by the misinterpretation of the thermodynamic fluctuations of the phase and the amplitude of the order parameter in superconductors with low charge carrier density [79,80,81] as the superconducting state. A strongly fluctuating Fermi sea is not an ordered superconducting condensate, where amplitude and phase coherence have been established across the entire sample. Based on this, it is incorrect to apply basic interpretations developed for superconducting condensate (at which a non-zero Meissner response should exist or, at least, zero resistance can be measured experimentally) to a system with strong local fluctuations in space and time [79,80,81], which are manifested as a drop of several percent in the resistance. 

Emery and Kivelson [80] proposed the temperature scale $T_{\varphi }$ for the phase fluctuations of the superconducting order parameter:

(10) $$T_{\varphi }=\frac{A\phi_{0}^{2}a}{4\pi \mu_{0}\lambda_{ab}^{2}\left(0\right)},$$

where a is the mean spacing between superconducting sheets for 2D superconductors and $a=\sqrt{\pi }\xi \left(0\right)$ for 3D superconductors, A=0.9 for 2D superconductors, and A=2.2 for 3D superconductors. 

Alternatively, Bulaevskii et al. [81] propose the following equation for the amplitude fluctuations of the superconducting order parameter:

(11) $$T_{amp}=\frac{\sqrt{\pi }}{3A}T_{\varphi }=\frac{\phi_{0}^{2}a}{12\sqrt{\pi }\mu_{0}\lambda_{ab}^{2}\left(0\right)},$$

If $T_{amp}$ and/or $T_{\varphi }$ are close to mean-field $T_{c}$, then thermal fluctuations are expected to break Cooper pairs, which causes the suppression of the observed superconducting transition temperature. For instance, in cuprates, strong phase fluctuations reduce the experimentally observed transition temperature, $T_{c}$, to well below its mean-field value, by more than 30% [80]. 

Returning now to the definition of the upper critical field, we can note that in several studies performed on perfect single-phase superconductors, the diamagnetic response was detected at $T_{c,dia-onset}$ which is below (and in many cases is well below) the temperature at which the resistance drops to zero; that is, $T_{c,dia-onset}\leq T_{c,zero}$ [35,82,83,84,85,86,87,88,89,90]. These rare but high-quality experimental reports provide additional evidence for the need to define the $B_{c2}\left(T\right)$ by at least the $\frac{R\left(T\right)}{R_{norm}\left(T\right)}\to 0$ criterion, which is the most accurate experimental value for the superconducting state collapse/emergence if only resistive measurements have been performed for the given sample. This $B_{c2}\left(T\right)$ definition has been implemented, but, again, in very rare studies [82,87,91,92,93] in comparison with the majority of studies, where $B_{c2}\left(T\right)$ was defined by the $0.5\leq \frac{R\left(T\right)}{R_{norm}\left(T\right)}<1$ criterion (see, for instance, [94,95]). 

It is interesting to note that Mandal et al. [87] defined $B_{c2}\left(T\right)$ as by $T_{c,dia-onset}$ (and for this definition, the derived $B_{c2}\left(0\right)=3.79\;T$) as well as by the $\frac{R\left(T\right)}{R_{norm}\left(T\right)}=0.5$ criterion (and for this definition, the derived $B_{c2}\left(0\right)=5.44\;T$) for bulk Zr_2_Ir single crystals. This result demonstrates that $B_{c2}\left(0\right)$ defined by the $\frac{R\left(T\right)}{R_{norm}\left(T\right)}=0.5$ criterion can be overestimated by a factor of 1.4. 

However, for Nd_0.775_Sr_0.225_NiO_2_ nickelate films, this difference is much larger, shown in Figure 4 in [23], where the difference between $B_{c2,perp}\left(T\right)$ defined by $\frac{R\left(T=6\;K\right)}{R_{norm}\left(T=6K\right)}=0.5$ and by $\frac{R\left(T=6\;K\right)}{R_{norm}\left(T=6\;K\right)}=0.01$ is approximately five times (the subscripts *perp* and *para* are for the direction of the applied magnetic field to the surface of the films). A much larger difference between $B_{c2}\left(T\right)$ defined by different $\frac{R\left(T\right)}{R_{norm}\left(T\right)}$ criteria was reported by Xiang et al. [22] for Nd_0.8_Sr_0.2_NiO_2_ thin films. These observed differences for nickelate films are much larger in comparison with bulk Zr_2_Ir [87] and bulk FeSe [71], which both exhibit a similar $T_{c}≅7\;K$ value and where the difference in $B_{c2}\left(T\right)$ defined by different $\frac{R\left(T\right)}{R_{norm}\left(T\right)}$ criteria is about 40%. 

Recently, independent direct confirmation that $T_{c,dia-onset}<T_{c,zero}$ in the nickelate films has been reported by Zeng et al. [45], who observed the inequality for films with thickness $5.5\;nm\leq d_{sc}\leq 15.2\;nm$. 

Outstanding differences between absolute $B_{c2}\left(T\right)$ values defined by different criteria were recently demonstrated in isotropic superconductor TaReSi by Shang et al. [96], and for anisotropic superconductor LaFeAsO by Zhigadlo et al. [97], while the vanishing of the diamagnetic response [96,97,98,99] remains the most accurate criterion to determine the upper critical field. In regard to the $B_{c2}\left(T\right)$ anisotropy, which is the topic of the study, different criteria of the $B_{c2}\left(T\right)$ definition lead to not only different $\gamma_{\xi }\left(T\right)$ values, but also to the change in the function trend, i.e., decrease/increase type [97]. 

Based on the above, in this report we define the $B_{c2}\left(T\right)$ at the lowest possible $\frac{R\left(T,B\right)}{R_{norm}\left(T,B=0\right)}$ criterion which can be applied to given experimental R(T,B) datasets (which depend on the signal/noise ratio and other real-world experimental issues). 

## 3. Results

### 3.1. La_0.8_Ca_0.2_NiO_2_ Film 

**Figure 1:**
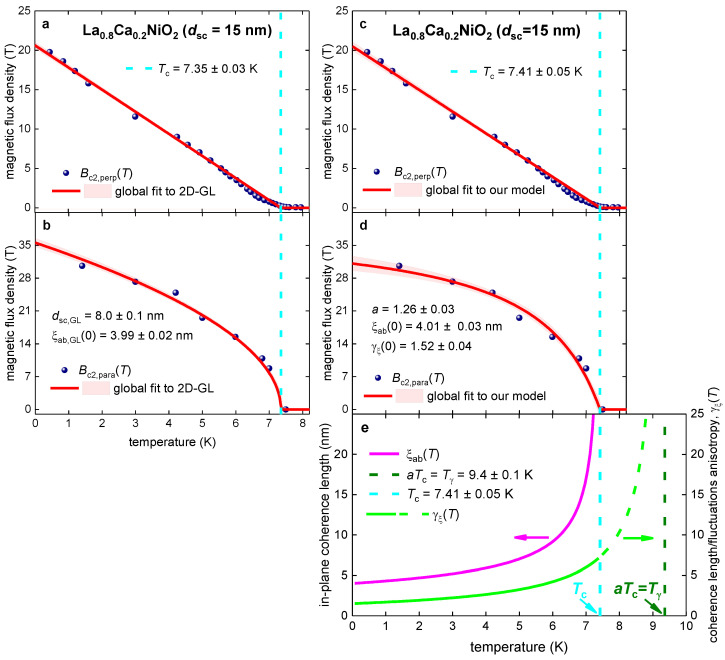
Global data fits to the 2D-GL model (Equations (1) and (2); panels (**a**,**b**)) and to our model (Equations (7)–(9); panels (**c**–**e**)) for the La_0.8_Ca_0.2_NiO_2_ film with physical thickness $d_{sc}=15\;nm$. The raw data were reported by Chow et al. [36]. Deduced parameters for (**a**) $B_{c2,perp}\left(T\right)$ and (**b**) $B_{c2,para}\left(T\right)$ are: $d_{sc,GL}=8.0\pm 0.1\;nm$, $T_{c}=7.35\pm 0.03\;K$, $\xi_{ab,GL}\left(0\right)=3.99\pm 0.02\;nm$. The goodness of fit is (**a**) 0.9975 and (**b**) 0.9907. Deduced parameters for (**c**) $B_{c2,perp}\left(T\right)$ and (**d**) $B_{c2,para}\left(T\right)$ data are: $T_{c}=7.41\pm 0.05\;K$, $\xi_{ab}\left(0\right)=4.01\pm 0.03\;nm$, $\gamma_{\xi }\left(0\right)=1.52\pm 0.04$, a=1.26±0.03. The goodness of fit is (**c**) 0.9963 and (**d**) 0.9832. (**e**) deduced $\xi_{ab}\left(T\right)$ and $\gamma_{\xi }\left(T\right)$. The 95% confidence bands are indicated by pink-shaded areas.

Chow et al. [36] reported $B_{c2,perp}\left(T\right)$ and $B_{c2,para}\left(T\right)$ datasets for La_0.8_Ca_0.2_NiO_2_ film (which has a physical thickness $d_{sc}=15\;nm$) defined by $\frac{R\left(T,B\right)}{R_{norm}\left(T,B=0\right)}=0.10$, $\frac{R\left(T,B\right)}{R_{norm}\left(T,B=0\right)}=0.50$, and $\frac{R\left(T,B\right)}{R_{norm}\left(T,B=0\right)}=0.90$ criteria. By following our discussion in the previous section, in Figure 1 we show reported $B_{c2,perp}\left(T\right)$ and $B_{c2,para}\left(T\right)$ datasets defined by the $\frac{R\left(T,B\right)}{R_{norm}\left(T,B=0\right)}=0.10$ criterion and global data fit to the 2D-GL model (Figure 1a,b; Equations (1) and (2)) and to our model (Figure 1c–e; Equations (7)–(9)). The quality of both fits (for which we used R-squared value [100]) is high, and the fitting parameters have low mutual dependence. However, deduced film thickness, $d_{sc,GL}=8.0\pm 0.1\;nm$, differs by nearly two times from physical film thickness $d_{sc}=15\;nm$, which is a manifestation of the general problem associated with utilization of Equations (1) and (2) for nickelate films [23], as we discussed above. 

It should be noted that the deduced $\xi_{ab,GL}\left(0\right)=3.99\pm 0.02\;nm$ is much smaller than any of the two film thicknesses (i.e., the physical thickness, $d_{sc}$, and the deduced thickness, $d_{sc,GL}$, from the fit):

(12) $$\xi_{ab,GL}\left(0\right)≅4\;nm<d_{sc,GL}=8\;nm$$

(13) $$\xi_{ab,GL}\left(0\right)≅4\;nm\;\ll d_{sc}=15\;nm$$

Considering that there is an expectation that $\xi_{c}\left(0\right)≲\xi_{ab}\left(0\right)$, Equations (12) and (13) imply that Equations (1) and (2) cannot be used to fit $B_{c2}\left(T\right)$ data for this film, because the film is not thin. 

Deduced coherence lengths from our model (Figure 1c–e) are $\xi_{ab}\left(0\right)=4.01\pm 0.03\;nm$ and $\xi_{c}\left(0\right)=\frac{\xi_{ab}\left(0\right)}{\gamma_{\xi }\left(0\right)}=2.64\;nm$. These values agree with our assumption that the film exhibits three-dimensional (3D) superconductivity:

(14) $$\xi_{c}\left(0\right)≅2.6\;nm<\xi_{ab}\left(0\right)≅4\;nm\;\ll d_{sc}=15\;nm,$$

The physical meaning of this part of the $\gamma_{\xi }\left(T\right)$ curve we discuss in the Discussion section; however, in short, it can be mentioned that the anisotropy should also exist for the phase and amplitude fluctuations of the order parameter above the transition temperature, $T_{c}$. Because all nickelates exhibit reasonably wide resistive transitions, similar to other unconventional superconductors (such as cuprates [80] and pnictides [101,102]), we can propose that the $a\times T_{c}$ value can be interpreted as the onset for the establishing of the anisotropy in superconducting fluctuations, $T_{\gamma }$, in the given material. 

Thus, our interpretation of the $T_{\gamma }=a\times T_{c}$ is based on the assumption that there is a universal temperature dependence for the anisotropy of the superconducting order parameter and of the fluctuations of this order above the superconducting transition, which, at least from the first glance, looks like a reasonable assumption. 

### 3.2. La_0.8_Sr_0.2_NiO_2_ Film 

**Figure 2:**
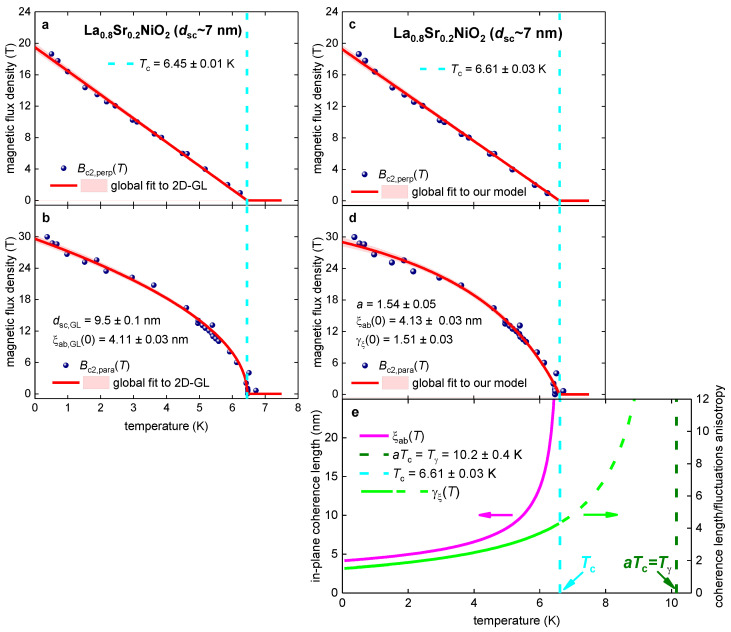
Global data fits to the 2D-GL model (Equations (1) and (2); panels (**a**,**b**)) and to our model (Equations (7)–(9); panels (**c**–**e**)) for the La_0.8_Sr_0.2_NiO_2_ film with physical thickness $d_{sc}\sim 7\;nm$. The raw data were reported by Wang et al. [103]. Deduced parameters for (**a**) $B_{c2,perp}\left(T\right)$ and (**b**) $B_{c2,para}\left(T\right)$ are: $d_{sc,GL}=9.5\pm 0.1\;nm$, $T_{c}=6.45\pm 0.01\;K$, $\xi_{ab,GL}\left(0\right)=4.11\pm 0.03\;nm$. The goodness of fit is (**a**) 0.9964 and (**b**) 0.9837. Deduced parameters for (**c**) $B_{c2,perp}\left(T\right)$ and (**d**) $B_{c2,para}\left(T\right)$ are: $T_{c}=6.61\pm 0.03\;K$, $\xi_{ab}\left(0\right)=4.13\pm 0.03\;nm$, $\gamma_{\xi }\left(0\right)=1.51\pm 0.03$, a=1.54±0.05. The goodness of fit is (**c**) 0.9959 and (**d**) 0.9897. (**e**) deduced $\xi_{ab}\left(T\right)$ and $\gamma_{\xi }\left(T\right)$. The 95% confidence bands are indicated by pink-shaded areas.

Wang et al. [103] reported $B_{c2,perp}\left(T\right)$ and $B_{c2,para}\left(T\right)$ datasets for La_0.8_Sr_0.2_NiO_2_ film (which has a physical thickness $d_{sc}\sim 7\;nm$) defined by the $\frac{R\left(T,B\right)}{R_{norm}\left(T,B=0\right)}=0.50$ criterion. In Figure 2, we show reported $B_{c2,perp}\left(T\right)$ and $B_{c2,para}\left(T\right)$ datasets and global data fit to the 2D-GL model (Figure 2a,b; Equations (1) and (2)) and to our model (Figure 2c–e; Equations (7)–(9)). The quality of both fits is high, and the fitting parameters have low mutual dependence. 

However, the deduced film thickness, $d_{sc,GL}=9.5\pm 0.1\;nm$, exceeds physical film thickness $d_{sc}=7\;nm$ (Figure 2a,b). In addition, the inequality of:

(15) $$\xi_{ab}\left(0\right)≅4\;nm<d_{sc}\sim 7\;nm<d_{sc,GL}=9.5\;nm$$

shows that the 2D-GL model cannot be used for the analysis (because the film is not sufficiently thin), despite a good fit quality. 

Deduced coherence lengths from the fit to our model (Equations (7)–(9)) are $\xi_{ab}\left(0\right)=4.13\pm 0.03\;nm$ and $\xi_{c}\left(0\right)=\frac{\xi_{ab}\left(0\right)}{\gamma_{\xi }\left(0\right)}=2.74\;nm$. These values agree with the assumption of our model that the film exhibits 3D superconductivity:

(16) $$\xi_{c}\left(0\right)≅2.7\;nm<\xi_{ab}\left(0\right)≅4.1\;nm<d_{sc}\sim 7\;nm,$$

In Figure 2c, we show the calculated temperature-dependent anisotropy of the coherence length: $\gamma_{\xi }\left(T\right)=\gamma_{\xi }\left(0\right)\frac{1}{1-\frac{1}{a}\times \frac{T}{T_{c}}}$, where all $\gamma_{\xi }\left(T\right)$ values for temperatures in the range of $T_{c}\leq T<a\times T_{c}\equiv T_{\gamma }$ are shown by the dashed line. 

### 3.3. Pr_0.8_Sr_0.2_NiO_2_ Film 

**Figure 3:**
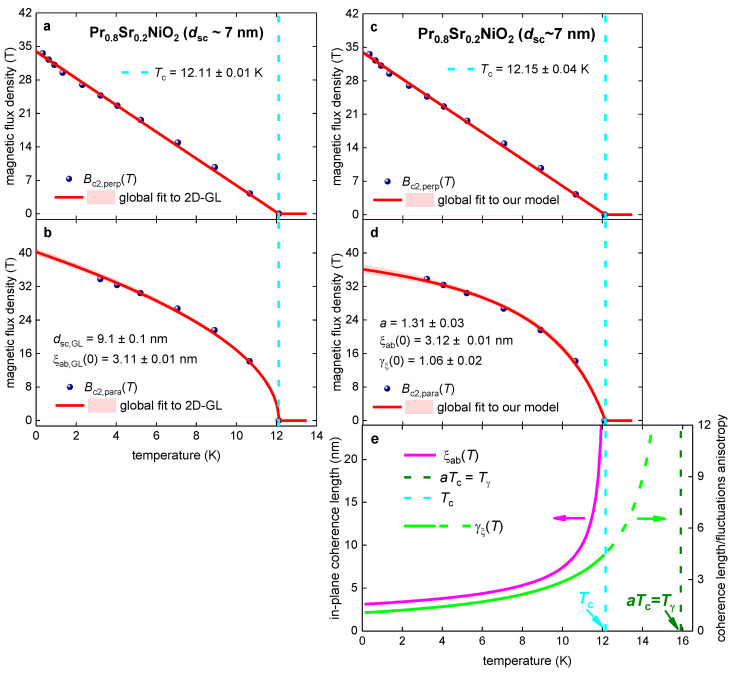
Global data fits to the 2D-GL model (Equations (1) and (2); panels (**a**,**b**)) and to our model (Equations (7)–(9); panels (**c**–**e**)) for the Pr_0.8_Sr_0.2_NiO_2_ film with physical thickness $d_{sc}\sim 7\;nm$. The raw data were reported by Wang et al. [103]. Deduced parameters for (**a**) $B_{c2,perp}\left(T\right)$ and (**b**) $B_{c2,para}\left(T\right)$ are: $d_{sc,GL}=9.1\pm 0.1\;nm$, $T_{c}=12.11\pm 0.01\;K$, $\xi_{ab,GL}\left(0\right)=3.11\pm 0.03\;nm$. The goodness of fit is (**a**) 0.9982 and (**b**) 0.9976. Deduced parameters for (**c**) $B_{c2,perp}\left(T\right)$ and (**d**) $B_{c2,para}\left(T\right)$ are: $T_{c}=12.15\pm 0.04\;K$, $\xi_{ab}\left(0\right)=3.12\pm 0.01\;nm$, $\gamma_{\xi }\left(0\right)=1.06\pm 0.02$, a=1.31±0.03. The goodness of fit is (**c**) 0.9984 and (**d**) 0.9985. (**e**) deduced $\xi_{ab}\left(T\right)$ and $\gamma_{\xi }\left(T\right)$. The 95% confidence bands are indicated by pink-shaded areas.

Wang et al. [103] reported $B_{c2,perp}\left(T\right)$ and $B_{c2,para}\left(T\right)$ datasets for Pr_0.8_Sr_0.2_NiO_2_ film (which has a physical thickness $d_{sc}\sim 7\;nm$) defined by the $\frac{R\left(T,B\right)}{R_{norm}\left(T,B=0\right)}=0.50$ criterion. In Figure 3 we show reported $B_{c2,perp}\left(T\right)$ and $B_{c2,para}\left(T\right)$ datasets and global data fit to the 2D-GL model (Figure 3a,b; Equations (1) and (2)) and to our model (Figure 3c–e; Equations (7)–(9)). The quality of both fits is high, and the fitting parameters have low mutual dependence. 

Overall, inequalities (similar to those obtained for the other nickelates (Equations (12)–(16)) were revealed for the Pr_0.8_Sr_0.2_NiO_2_ film:

(17) $$\xi_{ab}\left(0\right)≅4\;nm<d_{sc}\sim 7\;nm<d_{sc,GL}=9.5\;nm,$$

### 3.4. Nd_0.825_Sr_0.175_NiO_2_ Film 

**Figure 4:**
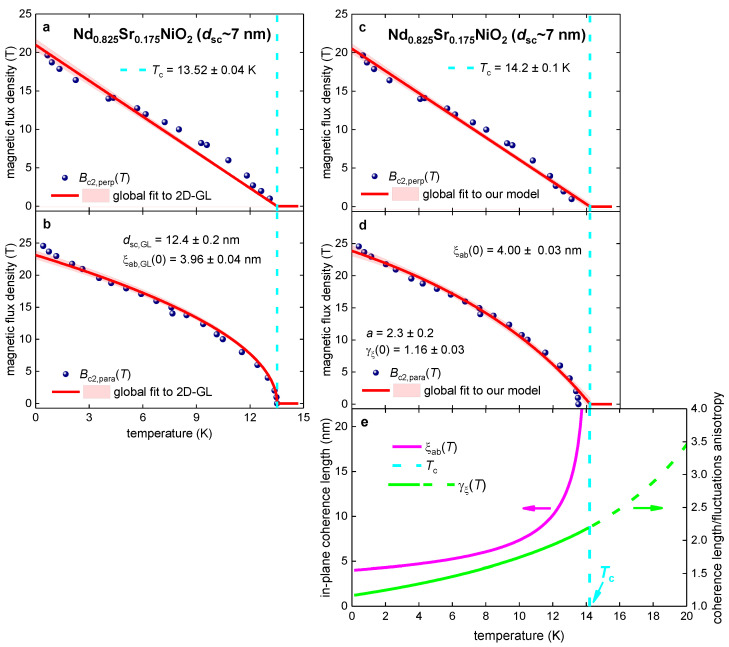
Global data fits to the 2D-GL model (Equations (1) and (2); panels (**a**,**b**)) and to our model (Equations (7)–(9); panels (**c**–**e**)) for the Nd_0.825_Sr_0.175_NiO_2_ film with physical thickness $d_{sc}\sim 7\;nm$. The raw data were reported by Wang et al. [103]. Deduced parameters for (**a**) $B_{c2,perp}\left(T\right)$ and (**b**) $B_{c2,para}\left(T\right)$ are: $d_{sc,GL}=12.4\pm 0.2\;nm$, $T_{c}=13.52\pm 0.04\;K$, $\xi_{ab,GL}\left(0\right)=3.96\pm 0.04\;nm$. The goodness of fit is (**a**) 0.9982 and (**b**) 0.9976. Deduced parameters for (**c**) $B_{c2,perp}\left(T\right)$ and (**d**) $B_{c2,para}\left(T\right)$ are: $T_{c}=14.2\pm 0.1\;K$, $\xi_{ab}\left(0\right)=4.00\pm 0.03\;nm$, $\gamma_{\xi }\left(0\right)=1.16\pm 0.03$, a=2.3±0.2. The goodness of fit is (**c**) 0.9846 and (**d**) 0.9900. (**e**) deduced $\xi_{ab}\left(T\right)$ and $\gamma_{\xi }\left(T\right)$. The 95% confidence bands are indicated by pink-shaded areas.

Wang et al. [103] reported $B_{c2,perp}\left(T\right)$ and $B_{c2,para}\left(T\right)$ datasets for Nd_0.825_Sr_0.175_NiO_2_ film (which has a physical thickness $d_{sc}\sim 7\;nm$) defined by the $\frac{R\left(T,B\right)}{R_{norm}\left(T,B=0\right)}=0.50$ criterion. In Figure 4, we show reported $B_{c2,perp}\left(T\right)$ and $B_{c2,para}\left(T\right)$ datasets and global data fit to the 2D-GL model (Figure 4a,b; Equations (1) and (2)) and to our model (Figure 4c–e; Equations (7)–(9)). The quality of both fits is high, and the fitting parameters have low mutual dependence. 

Deduced coherence lengths from the fit to our model (Equations (7)–(9)) are:

(18) $$\xi_{c}\left(0\right)≅3.4\;nm<\xi_{ab}\left(0\right)≅4.0\;nm<d_{sc}\sim 7\;nm,$$

which confirmed the 3D superconductivity of the Nd_0.825_Sr_0.175_NiO_2_ film. 

### 3.5. La_0.8_Sr_0.2_NiO_2_ Film 

**Figure 5:**
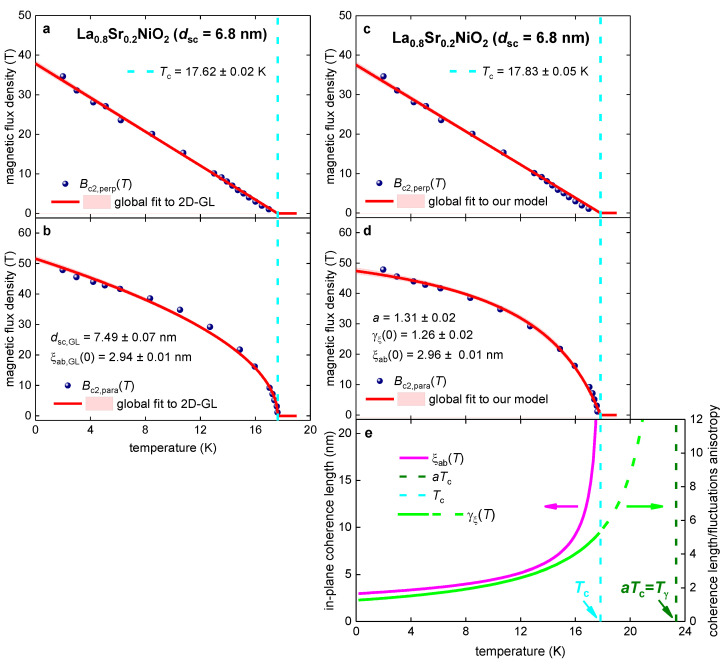
Global data fits to the 2D-GL model (Equations (1) and (2); panels (**a**,**b**)) and to our model (Equations (7)–(9); panels (**c**–**e**)) for the La_0.8_Sr_0.2_NiO_2_ film with physical thickness $d_{sc}=6.8\;nm$. The raw data were reported by Wei et al. [70]. Deduced parameters for (**a**) $B_{c2,perp}\left(T\right)$ and (**b**) $B_{c2,para}\left(T\right)$ are: $d_{sc,GL}=7.49\pm 0.07\;nm$, $T_{c}=17.62\pm 0.02\;K$, $\xi_{ab}\left(0\right)=2.94\pm 0.01\;nm$. The goodness of fit is (**a**) 0.9978 and (**b**) 0.9962. Deduced parameters for (**c**) $B_{c2,perp}\left(T\right)$ and (**d**) $B_{c2,para}\left(T\right)$ are: $T_{c}=17.83\pm 0.05\;K$, $\xi_{ab}\left(0\right)=2.96\pm 0.01\;nm$, $\gamma_{\xi }\left(0\right)=1.26\pm 0.02$, a=1.31±0.02. The goodness of fit is (**c**) 0.9965 and (**d**) 0.9976. (**e**) deduced $\xi_{ab}\left(T\right)$ and $\gamma_{\xi }\left(T\right)$. The 95% confidence bands are indicated by pink-shaded areas.

Now we return to the La_0.8_Sr_0.2_NiO_2_ compound, for which Wei et al. [70] recently reported the record high-superconducting transition temperature for nickelates. Wei et al. [70] also reported $B_{c2,perp}\left(T\right)$ and $B_{c2,para}\left(T\right)$ datasets defined by the $\frac{R\left(T,B\right)}{R_{norm}\left(T,B=0\right)}=0.01$, $\frac{R\left(T,B\right)}{R_{norm}\left(T,B=0\right)}=0.50$, and $\frac{R\left(T,B\right)}{R_{norm}\left(T,B=0\right)}=0.90$ criteria. Despite our understanding that $B_{c2}\left(T\right)$ should be defined by the lowest possible $\frac{R\left(T,B\right)}{R_{norm}\left(T,B=0\right)}$ criterion, in Figure 5 we analyze the $B_{c2}\left(T\right)$ data defined by the $\frac{R\left(T,B\right)}{R_{norm}\left(T,B=0\right)}=0.50$ criterion [70] to make it possible to make a comparison of deduced parameters for the La_0.8_Sr_0.2_NiO_2_ film in Section 3.2 (Figure 2). The film thickness is $d_{sc}\sim 6.8\;nm$ [70], which is practically the same as the one in the report by Wang et al. [103]. In Figure 5a,b we show $B_{c2}\left(T\right)$ data and global data fit to the 2D-GL model (Equations (1) and (2)) and to our model (Figure 5c–e); Equations (7)–(9)). 

Deduced coherence lengths from the fit to our model (Equations (7)–(9)) are:

(19) $$\xi_{c}\left(0\right)≅3.4\;nm<\xi_{ab}\left(0\right)≅4.0\;nm<d_{sc}\sim 7\;nm,$$

confirm the 3D superconductivity of the La_0.8_Sr_0.2_NiO_2_ film. 

In the following sections, we demonstrate that the high-quality fit of $B_{c2,perp}\left(T\right)$ and $B_{c2,para}\left(T\right)$ datasets to Equations (1) and (2) cannot be considered as evidence for 2D superconductivity because we obtained high-quality fits to Equations (1) and (2) for $B_{c2}\left(T\right)$ data for bulk iron-based superconductors (IBS). 

IBS were experimentally discovered by Hosono’s group [104,105] more than 15 years ago, and to the best of our knowledge, there has been no proposal for an analytical expression for the temperature-dependent coherence length anisotropy, $\gamma_{\xi }\left(T\right)$, to this family of superconductors. Here, we show that our 3D model (Equations (7)–(9)) can be extended to IBS materials. 

### 3.6. Bulk Tl_0.58_Rb_0.42_Fe_1.72_Se_2_

**Figure 6:**
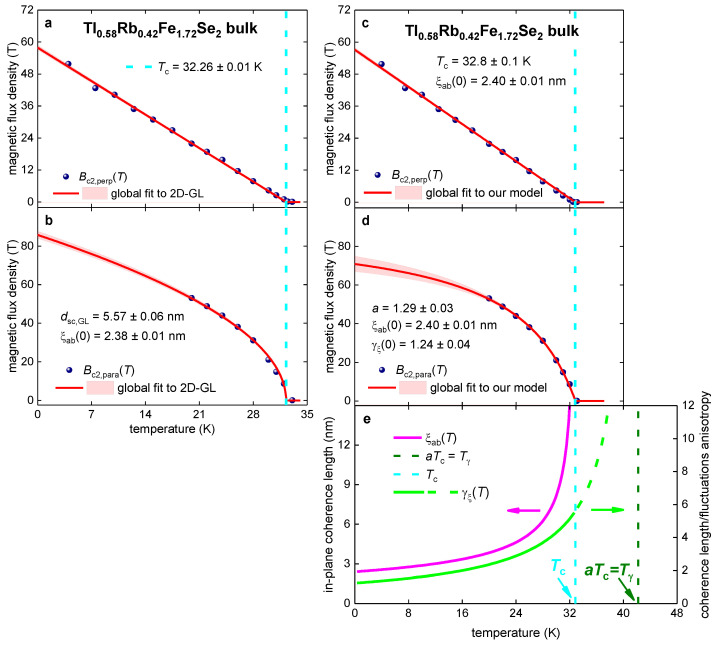
Global data fits to the 2D-GL model (Equations (1) and (2); panels (**a**,**b**)) and to our model (Equations (7)–(9); panels (**c**–**e**)) for bulk single crystals of Tl_0.58_Rb_0.42_Fe_1.72_Se_2_. The raw data reported by Jiao et al. [106]. Deduced parameters for (**a**) $B_{c2,perp}\left(T\right)$ and (**b**) $B_{c2,para}\left(T\right)$ are: $d_{sc,GL}=5.57\pm 0.06\;nm$, $T_{c}=32.26\pm 0.01\;K$, $\xi_{ab}\left(0\right)=2.38\pm 0.01\;nm$. The goodness of fit is (**a**) 0.9978 and (**b**) 0.9962. Deduced parameters for (**c**) $B_{c2,perp}\left(T\right)$ and (**d**) $B_{c2,para}\left(T\right)$ are: $T_{c}=32.8\pm 0.1\;K$, $\xi_{ab}\left(0\right)=2.40\pm 0.01\;nm$, $\gamma_{\xi }\left(0\right)=1.24\pm 0.04$, a=1.29±0.03. The goodness of fit is (**c**) 0.9984 and (**d**) 0.9994. The 95% confidence bands are indicated by pink-shaded areas.

Jiao et al. [106] reported $R\left(T,B_{perp}\right)$ and $R\left(T,B_{para}\right)$ datasets for bulk single crystals of Tl_0.58_Rb_0.42_Fe_1.72_Se_2_. These authors [106] derived extrapolative values for $B_{c2,perp}\left(T\right)$ and $B_{c2,para}\left(T\right)$ defined by the $\frac{R\left(T,B\right)}{R_{norm}\left(T,B=0\right)}\to 0.0$ and $\frac{R\left(T,B\right)}{R_{norm}\left(T,B=0\right)}\to 1.0$ criteria. Because these datasets of extrapolated values do not represent values measured in the experiment, in Figure 6 we analyze datasets, deduced by the $\frac{R\left(T,B\right)}{R_{norm}\left(T,B=0\right)}=0.50$ criterion, which represent the measured values. 

In Figure 6a,b, we fit $B_{c2,perp}\left(T\right)$ and $B_{c2,para}\left(T\right)$ datasets to Equations (1) and (2) to prove that high-quality fits to the 2D model can be obtained (and, even, “the thickness” of the superconductor, $d_{sc,GL}$, can be deduced) for $B_{c2}\left(T\right)$ data measured for bulk anisotropic superconductors. 

This implies (Figure 6a,b) that the thickness, $d_{sc,GL}$, of the “2D superconductor” can be deduced from $B_{c2,perp}\left(T\right)$ and $B_{c2,para}\left(T\right)$ datasets measured for bulk superconductors by utilizing the widely used [54,107] Equations (1) and (2) proposed by Ginzburg and Landau in the early 1960s [52,53]. 

Thus, we argue that Equations (1) and (2) are incorrect to use in data analysis because these equations represent reasonably flexible fitting functions, which can be used to smooth data for some superconductors. However, one parameter in these equations—that is, $d_{sc,GL}$—which exhibits a unit of length, does not have any physical meaning for bulk superconductors. 

This implies that the traditional interpretation (see, for instance, [23]) that the violation of the following equation:

(20) $$d_{sc}≅d_{sc,GL},$$

in thin film superconductors (including atomically thin superconductors) should indicate that there is a deep underlying physical effect (for instance, spin-orbit scattering [108,109]), cannot be found to be valid. 

In Figure 6c,d, we show the $B_{c2,perp}\left(T\right)$ and $B_{c2,para}\left(T\right)$ datasets for Tl_0.58_Rb_0.42_Fe_1.72_Se_2_ which were fitted to Equations (7)–(9). The fits are of high quality. The deduced parameters:

(21) a=1.29±0.03,

(22) $$\gamma_{\xi }\left(0\right)=1.24\pm 0.04,$$

are within the same ranges as those deduced for the nickelate films (Figure 1, Figure 2, Figure 3, Figure 4 and Figure 5). This is evidence that the nickelates exhibit 3D superconductivity. 

However, Figure 6 demonstrates that the 3D model (Equations (7)–(9)) can be extended to a broader range of superconductors, in particular, on bulk chalcogenides. To demonstrate this, in the next section we apply Equations (7)–(9) to another bulk single-crystal chalcogenide superconductor, Fe_1.11_Te_0.6_Se_0.4_ [110]. 

### 3.7. Bulk Fe_1.11_Te_0.6_Se_0.4_

**Figure 7:**
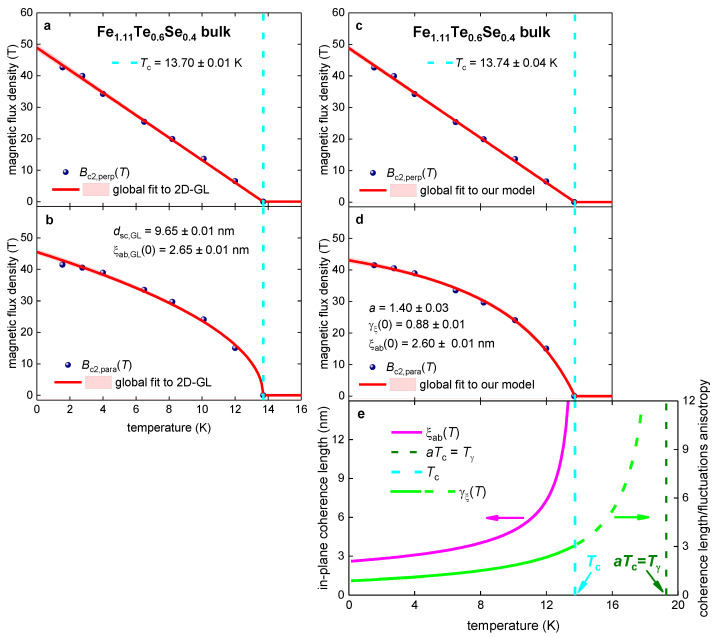
Global data fits to the 2D-GL model (Equations (1) and (2); panels (**a**,**b**)) and to our model (Equations (7)–(9); panels (**c**–**e**)) for bulk single crystals of Fe_1.11_Te_0.6_Te_0.4_. Raw data reported by Fang et al. [110]. Deduced parameters for (**a**) $B_{c2,perp}\left(T\right)$ and (**b**) $B_{c2,para}\left(T\right)$ are: $d_{sc,GL}=9.65\pm 0.01\;nm$, $T_{c}=13.70\pm 0.01\;K$, $\xi_{ab}\left(0\right)=2.65\pm 0.01\;nm$. The goodness of fit is (**a**) 0.9986 and (**b**) 0.9966. Deduced parameters for (**c**) $B_{c2,perp}\left(T\right)$ and (**d**) $B_{c2,para}\left(T\right)$ are: $T_{c}=13.74\pm 0.04\;K$, $\xi_{ab}\left(0\right)=2.60\pm 0.01\;nm$, $\gamma_{\xi }\left(0\right)=0.88\pm 0.01$, a=1.40±0.03. The goodness of fit is (**a**) 0.9988 and (**b**) 0.9989. The 95% confidence bands are indicated by pink-shaded areas.

Fang et al. [110] reported $B_{c2,perp}\left(T\right)$ and $B_{c2,para}\left(T\right)$ datasets for bulk single crystals of Fe_1.11_Te_0.6_Se_0.4_ [110] defined by the $\frac{R\left(T,B\right)}{R_{norm}\left(T,B=0\right)}=0.05$ criterion. In Figure 7a,b we fit $B_{c2,perp}\left(T\right)$ and $B_{c2,para}\left(T\right)$ datasets to Equations (1) and (2) to demonstrate that high-quality fits to the 2D-GL model can be obtained. 

Figure 7a,b show that “the thickness of the 2D superconductor”, $d_{sc,GL}$, of several nanometers (i.e., within a typical range usually deduced for thin film superconductors, including nickelates) can be deduced from the fit for this bulk anisotropic superconductor. 

In Figure 7c,d, the same $B_{c2,perp}\left(T\right)$ and $B_{c2,para}\left(T\right)$ datasets (as shown in Figure 7a,b, respectively), were fitted to Equations (7)–(9). The deduced parameters are in the expected ranges. 

However, the ground-state anisotropy of the coherence length is less than unity:

(23) $$\gamma_{\xi }\left(0\right)=0.88\pm 0.01<1.0$$

It should be noted that $\gamma_{\xi }\left(0\right)<1.0$ was reported for several iron-based superconductors, and this topic has been discussed (see, for instance Refs. [111,112]). 

### 3.8. Bulk KFe_2_As_2_

**Figure 8:**
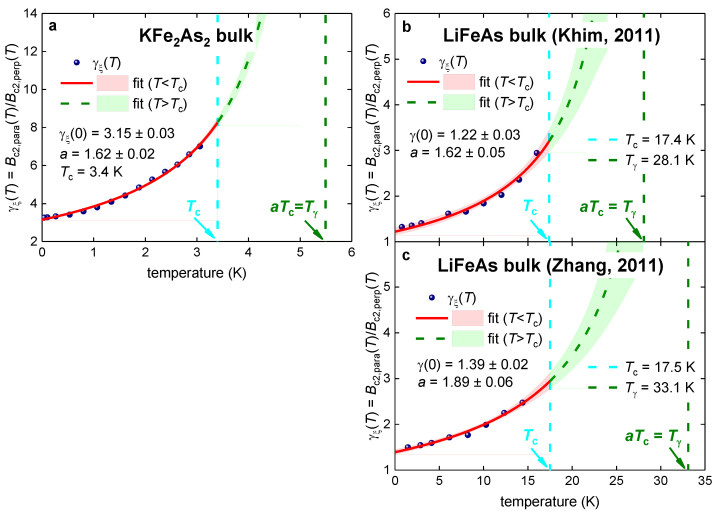
$\gamma_{\xi }\left(T\right)$ data and data fit to Equation (9) for single-crystal pnictides (**a**) KFe_2_As_2_ and (**b**,**c**) LiFeAs. Raw data reported by (**a**) Zocco et al. [113], (**b**) Khim et al. [114], and (**c**) Zhang et al. [115]. Deduced parameters are (**a**): $T_{c}=3.4\;\left(fixed\right)$, $\gamma_{\xi }\left(0\right)=3.15\pm 0.03$, a=1.62±0.02. The goodness of fit is 0.9963. (**b**) $T_{c}=17.4\;\left(fixed\right)$ [114], $\gamma_{\xi }\left(0\right)=1.22\pm 0.03$, a=1.62±0.05. The goodness of fit is 0.9777. (**c**) $T_{c}=17.5\;\left(fixed\right)$ [115], $\gamma_{\xi }\left(0\right)=1.39\pm 0.02$, a=1.89±0.06. The goodness of fit is 0.9871. The 95% confidence bands are indicated by pink-shaded areas.

Zocco et al. [113] reported $B_{c2,perp}\left(T\right)$, $B_{c2,para}\left(T\right)$, and $\gamma_{\xi }\left(T\right)=\frac{B_{c2,para}\left(T\right)}{B_{c2,perp}\left(T\right)}$ datasets for a bulk single-crystal KFe_2_As_2_ superconductor. In Figure 8a, the reported $\gamma_{\xi }\left(T\right)$ is fitted to Equation (9) (for this fit, we fixed the transition temperature to the value observed in the experiment, $T_{c}=3.4\;\left(fixed\right)$). 

The fit is of high quality and has low mutual parameters dependence. The deduced parameters (Figure 8) are within the ranges reported above for nickelates and iron-based superconductors. 

### 3.9. Bulk LiFeAs 

Khim et al. [114] and Zhang et al. [115] reported $B_{c2,perp}\left(T\right)$, $B_{c2,para}\left(T\right)$, and $\gamma_{\xi }\left(T\right)=\frac{B_{c2,para}\left(T\right)}{B_{c2,perp}\left(T\right)}$ datasets for a bulk single-crystal pnictide LiFeAs superconductor. In Figure 8b,c we show the fits of the reported $\gamma_{\xi }\left(T\right)$ to Equation (9) (for this fit, we set the transition temperature to the value observed in the experiments). 

The fits are of a high quality and have low parameters dependence. The deduced parameters for two datasets reported by independent research groups are close to each other, within acceptable levels of parameter differences. 

## 4. Discussion

**Figure 9:**
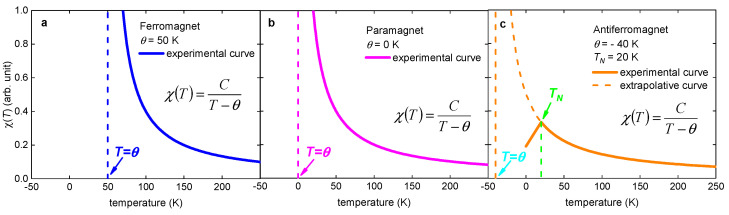
Schematic representation of the DC magnetic susceptibility χ(T) obeys the Curie–Weiss law for (**a**) ferromagnetic, (**b**) paramagnetic, and (**c**) antiferromagnetic materials. θ is Curie–Weiss temperature (the analogue value in our model is $T_{\gamma }\equiv aT_{c}$), C is Curie constant (the analogue value in our model is $\left(aT_{c}\right)\times \gamma_{\xi }\left(0\right)$), $T_{N}$ is Neel temperature (the analogue value in our model is $T_{c}$).

The physical origin of our model (which is primarily based on Equation (9)) can be understood based on an analogy with the temperature-dependent DC magnetic susceptibility, χ(T), in antiferromagnetic materials [116,117]. The temperature-dependent χ(T) in any material obeys the Curie–Weiss law (Figure 9):

(24) $$\chi \left(T\right)=\frac{C}{T-\theta },$$

where θ is Curie–Weiss temperature, and *C* is the Curie constant. 

In the schematic representations of Equation (24) in Figure 9, there are three types of magnetic materials that primarily depend on the sign of the Curie–Weiss temperature:

1. θ>0 K for ferromagnetic materials;
2. θ=0 K for paramagnetic materials;
3. θ<0 K for antiferromagnetic materials.

To be consistent with the form of Equation (24), we can rewrite Equation (9):

(25) $$\gamma \left(T\right)=\frac{aT_{c}\gamma \left(0\right)}{aT_{c}-T}=-\frac{aT_{c}\gamma \left(0\right)}{T-aT_{c}}=-\frac{C}{T-\theta },$$

where $C=aT_{c}\gamma \left(0\right)$, and $\theta =aT_{c}$. 

Despite the negative sign (in K units) of the Curie–Weiss temperature, θ, for antiferromagnetic materials, this value represents one of the fundamental constants of the antiferromagnet, which quantifies the strength of the antiferromagnetic interaction in the material. 

In antiferromagnetic materials, the χ(T) would obey the Curie–Weiss law down to very low temperatures, T→0 K (Figure 9c). However, at the Neel temperature, $T_{N}>0\;K$, a phase transition occurs, and the χ(T) does not obey the Curie–Weiss law at $T<T_{N}$. 

In our model, $\gamma_{\xi }\left(T\right)$ would obey the Equation (9) up to high temperatures, $T\to aT_{c}\equiv T_{\gamma }$; however, at $T=T_{c}$, a superconductor–normal state phase transition occurs, and $\gamma_{\xi }\left(T\right)$ becomes undefined at $T>T_{c}$. However, the latter does not mean that $aT_{c}\equiv T_{\xi }$ does not represent any physical value for the material, and our current interpretation of this value is that $T=aT_{c}\equiv T_{\gamma }$ represents the threshold temperature for the appearance of the anisotropy in the fluctuations of the order parameter in a superconductor. 

It should also be stressed that our model (Equations (7)–(9)) utilizes the simplistic GL expression for the temperature-dependent in-plane coherence length:

(26) $$\xi_{ab}\left(T\right)=\frac{\xi_{ab}\left(0\right)}{\sqrt{1-\frac{T}{T_{c}}}},\;$$

Perhaps it would be more accurate to use the Werthamer–Helfand–Hohenberg (WHH) theory [118] or its advanced version developed for two-band superconductors by Gurevich [119]. This type of advanced analysis, in conjunction with high-field experimental studies, has been implemented in several studies on IBS [120,121] and nickelates [23]. 

However, the high flexibility of primary WHH functions (exhibited several parameters, especially for the two-band model), and the nonexistence of WHH functions for $T>T_{c}$, makes it impossible to extract a simple analytical expression, similar to Equation (9), for the temperature dependence of the coherence length anisotropy, γ(T), which we propose herein. That is, the fact that:

(27) $${\xi_{ab}\left(T\right)|}_{T\to T_{c}}\to \infty ,\;$$

(28) $${\xi_{c}\left(T\right)|}_{T\to T_{c}}\to \infty ,\;$$

does not exclude the following:

(29) $$\gamma_{\xi }\left(T\right)={\frac{\xi_{ab}\left(T\right)}{\xi_{c}\left(T\right)}|}_{T\to T_{c}}\neq \infty .$$

The rigorous mathematical expression for the primary message of this study is as follows:

(30) $$\forall \frac{T}{aT_{c}}<1,\;\exists \;\gamma_{\xi }\left(T\right)\in {\mathbb{R}}_{>0},\;$$

where the standard mathematical symbols are used. 

In this regard, the simple heuristic expression for $\gamma_{\xi }\left(T\right)$ (proposed herein (that is, Equation (9))) might be modified to be more accurate (and unfortunately, more complicated); however, our primary message that the anisotropy of the thermodynamic fluctuations in anisotropic superconductors (exhibiting low charge-carrier density) is established at some temperature $T_{\gamma }\equiv aT_{c}$ (a>1) above the entire superconducting transition temperature, $T_{c}$, should remain unchanged. 

The hypothetical analogy between temperature dependencies for some primary physical quantities for the antiferromagnetism and the superconductivity phenomena proposed herein (Figure 9) does not have any deeper meaning, except that the shape of the temperature dependencies can be similar. However, perhaps there is a much deeper physical ground between these two physical phenomena, because recently Fowlie et al. [37] showed that nickelates (regardless of the rare earth ion or doping) exhibit an intrinsic magnetic ground state arising from the short-range antiferromagnetic order of the nickel sublattice. Considering that the interplay/relation between the magnetism and the superconductivity in other unconventional superconductors has been discussed for decades [122,123,124], there is a chance that deeper relations can be revealed by further theoretical and experimental investigations. 

All superconductors exhibit the second fundamental characteristic length, which is the London penetration depth, λ(T). In anisotropic superconductors, the λ(T) also has two components, which are in-plane London penetration depth, $\lambda_{ab}\left(T\right)$, and out-of-plane London penetration depth, $\lambda_{c}\left(T\right)$. We expect that the same approach, to that described herein for $\gamma_{\xi }\left(T\right)$, should be applied for the temperature-dependent London penetration depth anisotropy, $\gamma_{\lambda }\left(T\right)=\frac{\lambda_{ab}\left(T\right)}{\lambda_{c}\left(T\right)}$ [72,125,126,127]. However, the discussion of this topic is far beyond the framework of the current study. 

## 5. Conclusions

In this study, we analyze the temperature dependence of the upper critical field anisotropy and the coherence length anisotropy, $\gamma_{\xi }\left(T\right)$, in nickelate superconductors for which we propose a simple heuristic expression (Equation (9)) within the 3D Ginzburg-Landau model. The proposed expression for $\gamma_{\xi }\left(T\right)$ (Equation (9)) is also applicable to some chalcogenide and pnictide superconductors. 

Overall, we show that 2D Ginzburg–Landau model, proposed by Tinkham and co-workers [52,107] (and which was the dominant model to describe the upper critical field in thin film superconductors for more than 50 years) is not a unique approach to describe experimental upper critical field data in thin film superconductors, and alternative 3D models can be used to analyze experimental data for thin film superconductors (which satisfy the condition $\xi_{c}≲d_{sc}$). 

## Data Availability

Not applicable.
